# Regenerative Medicine Therapy in Malaysia: An Update

**DOI:** 10.3389/fbioe.2022.789644

**Published:** 2022-04-26

**Authors:** Siti A. M. Imran, M. Haikal Aiman M. Hamizul, Ahmad Amin Noordin Khairul Bariah, Wan Safwani Wan Kamarul Zaman, Fazlina Nordin

**Affiliations:** ^1^ Centre for Tissue Engineering and Regenerative Medicine, Faculty of Medicine, Universiti Kebangsaan Malaysia, Kuala Lumpur, Malaysia; ^2^ School of Dental Sciences, Universiti Sains Malaysia, Kampus Kesihatan Kubang Kerian, Kelantan; ^3^ Department of Biomedical Engineering, Faculty of Engineering, Universiti Malaya, Kuala Lumpur, Malaysia

**Keywords:** regenerative medicine therapy, cell therapy, stem cells, tissue engineering, biomaterials, clinical trials, Malaysia, Asia

## Abstract

Regenerative medicine is a field in medicine that relates to the ability to correct congenital anomalies and to repair or replace tissues and organs that have been destroyed by age, disease, or trauma. To date, promising preclinical and clinical data supported the possibility of using regenerative medicine to treat both chronic diseases and acute insults, as well as maladies affecting a wide range of organ systems and contexts, such as dermal wounds, cardiovascular diseases and traumas, cancer treatments, and more. One of the regenerative medicine therapies that have been used widely is stem cells. Stem cells, especially mesenchymal and hematopoietic stem cells, play an important role in treating chronic diseases, such as leukemia, bone marrow, autoimmune disease, and urinary problems. Despite considerable advancements in stem cell biology, their applications are limited by ethical concerns about embryonic stem cells, tumor development, and rejection. Nevertheless, many of these constraints, are being overcome, which could lead to significant advancements in disease management. This review discusses the current developments and advancements of regenerative medicine therapy (RMT) advancements in Malaysia compared to other Asian countries. The limitations in the application of RMT are also highlighted.

## 1 Introduction

Regenerative medicine therapy (RMT) is a process of replacing and regenerating human cell tissues and organs to restore or establish normal function. This field holds the promise of regenerating tissues and organs in the body by replacing damaged tissues or by stimulating growth mechanisms for tissue and organ healing (Mao and Mooney 2015). In RMT, tissues and organs are grown in the laboratory before they are safely implanted when the body is unable to heal (Bioengineering NIOBIA 2020). The tissues are retrieved in two ways: through cell culture and from cells that have been extracted from humans or animals (Fuchs 2012). To grow stem cells, scientists first extract samples from adult tissue or an embryo (Bioengineering NIOBIA 2020). The cells are then placed in a controlled culture where they will divide and proliferate but not specialized further. A stem-cell line is a collection of stem cells that are dividing and replicating in a controlled environment (Bioengineering NIOBIA 2020). Regenerative medicine strives to make it easier to repair or regenerate damaged tissues or organs (Bioengineering NIOBIA 2020).

**FIGURE 1:**
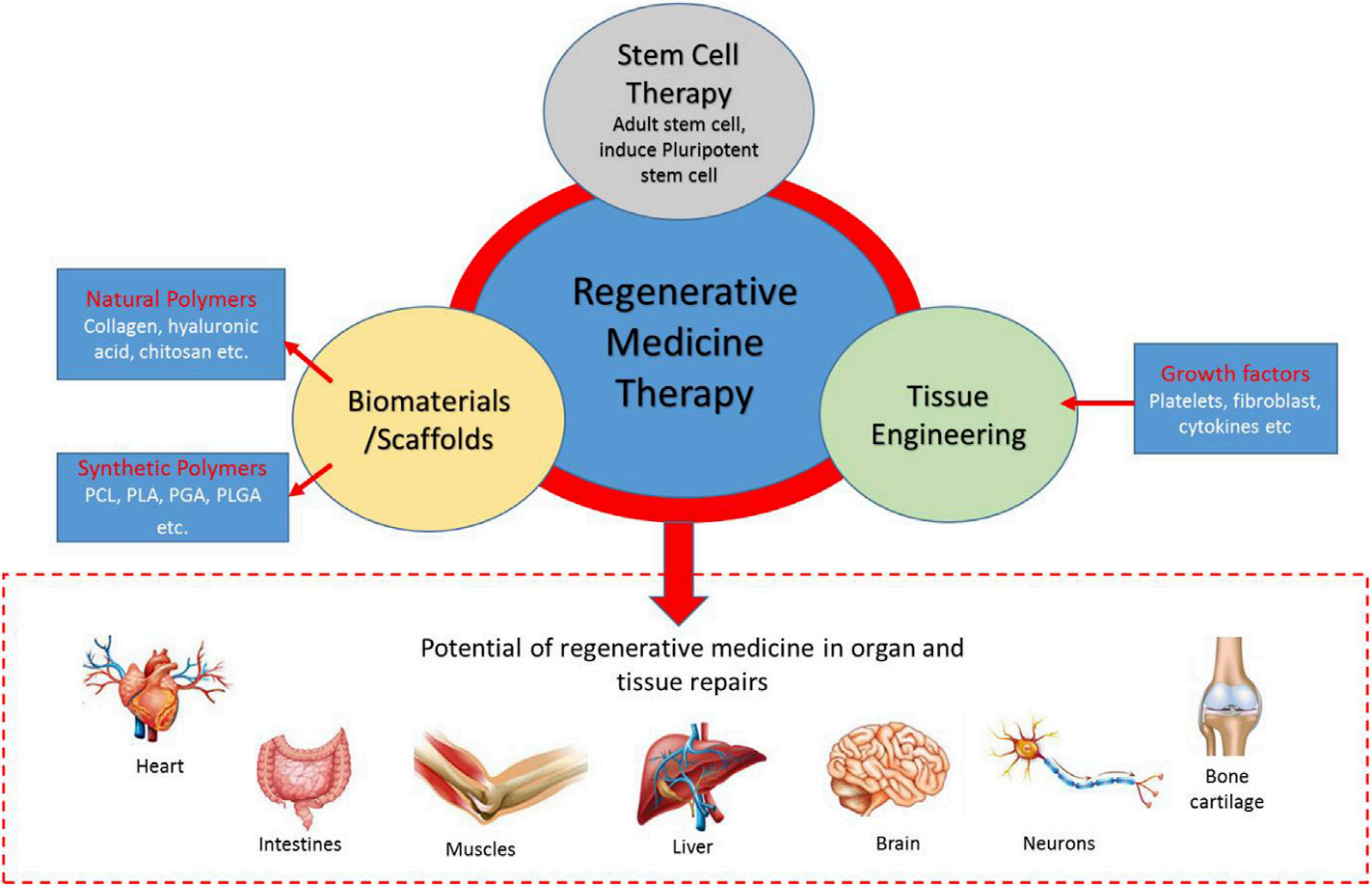
The regenerative medicine therapy (RMT) triad. Stem cell therapy, biomaterials/scaffolds, and tissue engineering are components of regenerative medicine in organ/tissue repairs.

A healthy human tissue, such as the liver, could naturally regenerate if destroyed either by disease or injury (Krafts 2010). Such tissue can return to its original size and function but not its original shape (Krafts 2010), however, the tissues do not regenerate in some cases (Krafts 2010). This is where regenerative medicine comes into play as it aims to promote tissue regeneration in the body or use tissue engineering for tissue replacements. As shown in Figure 1, RMT involves cell therapy, tissue engineering, and biomaterials. Biomaterials, also known as scaffolds, consist of natural and synthetic polymers. Meanwhile, common growth factors are critical molecules for tissue repair and regeneration, such as platelet, fibroblast, and vascular endothelial cells. Adult stem cells (induced pluripotent stem cells) are living cells that originate from adult somatic cells, and their potential use in personalized regenerative medicine has been demonstrated (Stoddard-Bennett and Pera 2018). These components are important because of the potential of regenerative medicine in organ and tissue repairs, such as brain, liver, skin, bone, cartilage, and nerve cell, and their applications in clinical settings.

Among the RMTs, stem cells transplantation, cellular therapy, and platelet-rich plasma (PRP) are the most prominent medical therapies being offered to patients. Others such as fibroblast cells, stem cell, and their cellular matrix, including PRP are used in anti-aging therapies. These diverse benefits depict that the potential of RMT can be further explored and implemented. Hence, this review is important to highlight and compare the progress and update of current RMT treatments or services including clinical trials in Malaysia with other developed Asian countries.

Malaysia has grown and become a strong competitor in the global health and medical tourism sector, which is now identified as a center of medicine in the region. There are two categories of health tourism offered in Malaysia: medical tourism and wellness programs. Patients may choose medical treatments and stay on the recuperating period, or they may come for a holiday and explore the various wellness programs in over 14 different states in Malaysia. With more than 35 well-established private hospitals, Malaysia offers a wide choice of state-of-the-art in-patient facilities and comfortable accommodation ranging from private rooms to luxury suites, a fine variety of meals, and highly qualified and trained nurses at a reasonable cost. Additionally, these hospitals are certified and internationally recognized quality standards, such as MS ISO 9002, and accredited by the Malaysian Society for Quality of Health.

Malaysia has become a preferred health tourism destination for many abroad patients. It offers competitive and affordable pricing and favorable exchange rate, highly qualified, experienced, and skilled consultants. Malaysia is ranked first in medical tourism compared to other countries as it attracted approximately 1.3 million medical tourists in 2019. Although the diverse culture and religion could be barriers in the implementation and practice of RMT, however, the unique entity and tolerant multi-racial and multi-cultural environment in Malaysia has successfully accommodated patients of different cultures and religions.

### 1.1 Stem Cell Therapies

Stem cells are repair units of the body that provide a central function in the regeneration of organ tissues. The main function of stem cells is to replenish dying cells and regenerate damaged tissues, which have the potential to be used as treatments for many diseases, such as cardiovascular diseases and different types of cancer. These events have heightened the hope of achieving stem cell-based replacement therapy in medicine. The three types of stem cells include embryonic stem cells, adult stem cells, and perinatal stem cells. For embryonic stem cells, the embryo (at this stage, called a blastocyst) is isolated from inner cell mass (ICM) that can form all the specialized tissues that make up the human body on days 3–5 following fertilization and before implantation. Embryonic stem cells were first isolated from mice’s ICM from preimplantation mouse blastocysts *in vitro* (Evans and Kaufman 1981; Martin 1981). These cells, termed Embryonic Stem Cells (ESCs), could self-renew indefinitely in culture, while being able to be differentiated into cells derived from all three germ layers, except for the placenta. Human embryonic stem cells (hESC) are obtained from the inner cell mass of an *in vitro* fertilized embryo that has been donated for research purposes after receiving informed consent. These pluripotent stem cells, which can transform into any cell type, are only found in the early developmental stages (Mehta 2014).

There are four types of adult stem cells, namely, the Hematopoietic Stem Cells (HSCs), Mesenchymal Stem Cells (MSCs), Neural Stem Cells (NSCs), and Epithelial Stem Cells (EpiSCs). ESCs are pluripotent, meaning they can produce every cell type in the fully formed body except the placenta and umbilical cord. These cells are extremely significant since they provide a sustainable resource for research into normal development and disease, as well as drug and therapy testing. Adult stem cells are assumed to be tissue-specific, with the ability to develop only into offspring cells of the tissues they originated from (Fang et al., 2004). Furthermore, adult stem cells have a limited ability to differentiate; they can be either be multipotent or unipotent. Firstly, HSCs are the rare and multipotent cells that reside in the bone marrow (BM), and they are responsible for the life-long production of all types of mature blood cells (Bryder et al., 2006). HSCs are cells that give rise to other blood cells via a process called hematopoiesis. Hematopoiesis refers to the production of all mature blood cells, and hematopoietic stem cells can undergo self-renewal and replenish all blood cell types (Seita and Weissman 2010).

HSCs containing grafts are being used in the treatment of blood cell diseases, such as leukemia and autoimmune disorders. MSCs can be found in the bone marrow and can be isolated in the tissues, including lung, cord blood, peripheral blood, and fallopian tube (Klingemann et al., 2008). This stem cell can differentiate to form adipocytes, bone cells, cartilage, muscle cells, neural cells, and skin cells. The umbilical cord tissue, molar teeth, body fat, and amniotic fluid are examples of the common sources for MSCs. Researchers have discovered the ability to augment a person’s stem cell count through transplantation with younger and more capable cells by collecting MSCs from donated cord tissue and increasing them to larger quantities (Ullah et al., 2015). MSCs are a popular source for regenerative medicine given their ability to respond to mechanical environments, including substrate mechanics and extrinsic mechanical stimuli (Steward and Kelly 2015). Perinatal stem cells are stem cells in amniotic fluid and umbilical cord, which could differentiate into specialized cells, such as adult stem cells (Zakrzewski et al., 2019). Stem cells have been widely used in RMT applications worldwide (Zakrzewski et al., 2019). Their ability to self-renew and differentiate to various cell types present an exceptional potential and possibility for RMT applications. The most common stem cell therapy widely used in RMT is HSCs transplantation (Khaddour et al., 2021). Meanwhile, bone marrow, peripheral blood, and umbilical cord blood are the most common sources of the target cells in HSCs transplantation (Hordyjewska et al., 2015).

### 1.2 Tissue Engineering

Tissue engineering is a biomedical engineering discipline that restores, maintains, improves, or replaces biological tissues using a combination of cells, engineering, materials technologies, and appropriate biochemical and physicochemical parameters (Bioengineering NIOBIA 2020). Tissue engineering evolved from the biomaterial field, which refers to the practice of combining scaffolds, cells, and biologically active molecules into functional tissues. Tissue engineering aims to assemble functional constructs that restore, maintain or improve damaged tissue or whole organs (Bioengineering NIOBIA 2020). There are three components of tissue engineering which are; 1) reparative cells that can form functional matrices, 2) appropriate scaffold for transplantation, 3) bio-reactive molecules that promote and choreograph the production of the desired tissue. These three components can be employed separately or in combination to regenerate organs or tissues (de Isla et al., 2010). The scaffolds are typically made up of a biodegradable polymer, extracellular matrix (ECM), and growth factors, and they serve as a skeleton for cells to fill and eventually grow into three-dimensional tissues (Kim et al., 2016). The extracellular matrix is made and secreted by groups of cells, which serves as a relay station for several signaling molecules in addition to supporting the cells (Assunção et al., 2020). Resultantly, cells receive messages from a variety of sources emerging from the immediate environment. Each signal can set off a series of events that determine the cell’s fate (Levin et al., 2017). These processes have been successfully managed by researchers to repair damaged tissues or even build new ones by studying how individual cells respond to signals, interact with their environment, and organize into tissues and organisms (de Isla et al., 2010).

In addition to the extracellular matrix, a few growth factors components exist in tissue engineering. Growth factors are important chemicals for tissue regeneration and repair (Zheng et al., 2019). As a result, recombinant growth factors have sparked extensive interest in the field of regenerative medicine. While the use of growth factors to enhance tissue healing has shown encouraging outcomes in preclinical studies, clinical success is not guaranteed. The translation of growth factors is frequently hindered by their short half-life, fast dispersion away from the delivery site, and low cost-effectiveness (Ren et al., 2020). Future growth factor-based regenerative therapy solutions may benefit from a more holistic approach to tissue regeneration, as it is now clear that the immune system plays a vital role in the regenerative process. Future efforts could profit from the introduction of growth factors and immunomodulators, or the production of multifunctional fusion proteins that could promote morphogenesis while influencing the immune system. Furthermore, most of the delivery systems discussed here are designed to improve the release and stability of growth factors at the delivery site (Julier et al., 2017; Larouche et al., 2018).

### 1.3 Biomaterials

Biomaterials are any materials that interact with biological systems, whether a substance, surface, or structure. Biomaterials can be found in nature or created in the lab with the use of metallic components, polymers, ceramics, and composite materials (Julier et al., 2017). Biomaterial-based medical devices are frequently used to replace or augment natural functions. A few examples include heart valves, hip replacements, and materials used in dentistry and surgery (Lam and Wu 2012). Biomaterials used to create porous scaffolds for tissue engineering can be divided into two categories based on their origins: natural and synthetic biomaterials (Lam and Wu 2012). Porous scaffolds can be made from naturally existing biomaterials that can be obtained from their natural sources (Chan and Leong 2008). These materials can be in their natural state, such as ECM from allografts and xenografts, or in the form of smaller building blocks, such as inorganic ceramics (i.e., calcium phosphates) and organic polymers (i.e., proteins, polysaccharides, lipids, and polynucleotides) among others. Natural biomaterials are often highly biocompatible, allowing cells to connect and proliferate with high viability. On the other hand, natural materials have limited physical and mechanical stability, making them unsuitable for some load-bearing applications (Chan and Leong 2008). This explains why researchers working with natural biomaterials resort to creating solutions that improve and reinforce the mechanical properties and stability of natural biomaterials. Inorganic biomaterials, such as bioglasses and organic biomaterials (i.e., synthetic polymers) are two types of synthetic biomaterials. Synthetic biomaterials are thought to have more regulated physical and mechanical properties and could be tailored for both soft and hard tissues. Biocompatibility becomes a serious concern for synthetic biomaterials as cells may have difficulty attaching to and growing on these materials (Chan and Leong 2008). Hence, a variety of techniques for altering the surface and bulk properties have been developed to increase biocompatibility (Morra and Cassinelli 2006).

Biomaterials are crucial in regenerative medicine, particularly in the replacement of injured tissues and organs, as well as the treatment of chronic diseases to restore normal body function (Mao and Mooney 2015). Maintaining the optimal environment for the cells to grow remains the main challenge of using biological products, such as cells in therapy. Therefore, biomaterials are being used to overcome these challenges. The function of biomaterials is to regulate cellular responses including cell-cell and cell-matrix interaction. It also acts as a scaffold to provide structural support for both cell adhesion and tissue development. Examples of biomaterials that can be used are metals, ceramics, plastics, glass, lung cells, and tissues. They could also be re-engineered into machined and molded parts, creating foams, fibers, and fabrics for medical products. Biomaterials applications may include hip joint replacement, heart valve transplantation, dental implants, and contact lenses (Choi 2019).

### 1.4 Aesthetic Applications of Regenerative Medicine Therapy

In addition to the clinical use of RMT in disease treatments, RMT is also known for its aesthetic applications. Most people know RMT to be an innovative therapy that allows the body to regenerate aging cells, tissues, and organs. Given its relative accessibility, the skin is a particularly appealing organ for the application of innovative regenerative therapies. Stem cells and PRP have piqued attention among these treatments due to their therapeutic potential in scar reduction, anti-aging, and alopecia treatment. Specifically, alopecia is a condition that occurs when the immune system attacks the hair follicles, causing hair loss in small spots that might be difficult to be visualized with an unaided eye. Aesthetic application of RMT is always associated with self-renewal and adaptability, which are two of the most important characteristics of stem cells.

Another important characteristic of stem cells is plasticity, which is the ability of adult tissue-specific stem cells to switch to new identities (Ramaswamy Reddy et al., 2018). Stem cells, when combined with anti-aging genes, can form a complex barrier that protects against the consequences of aging. Increased wear and tear on the body’s natural stem cells causes cellular damage and speeds up the aging process (Nguyen et al., 2019). Injecting “youthful” stem cells into the human body can regenerate existing cells, allowing the body to age gracefully and even reverse some aging symptoms. However, to date, there is inadequate clinical evidence that supports the use of stem cells in helping people age gracefully. One of the renowned aesthetic applications of RMT as an anti-aging treatment is for hair loss. Hair loss is caused by androgenic alopecia that may affect both men and women. Male-pattern baldness is another name for this problem in men. Hair loss is more common in men who transform testosterone into the more powerful metabolite dihydrotestosterone in hair follicle cells (DHT) (Rathnayake and Sinclair 2010). DHT binds to the androgen receptor in hair follicles, reducing cyclic AMP levels. The concentration of AMP (cAMP) in the cell slows down the metabolism of sugar in hair follicles and shortens the time it takes for hair follicles to grow by suppressing energy supply (Rathnayake and Sinclair 2010). As a result, the hair follicle gradually becomes more active as the resting phase of the hair becomes shorter and thinner.

Another RMT that has been used aesthetically is PRP. PRP therapy is the process of extracting platelets from the blood and centrifuging them to significantly higher concentrations (Dhurat and Sukesh 2014). Plasma is a clear, colorless liquid that is devoid of cells. Platelets are cells that are shaped like dinner plates, therefore their name. Like red blood cells and most white blood cells, platelets are produced in the bone marrow. Platelets are produced by megakaryocytes, which are extraordinarily massive bone marrow cells. Megakaryocytes undergo a process of fragmentation as they mature into gigantic cells, resulting in the discharge of over 1,000 platelets per megakaryocyte. The function of platelets is to help the body form a clot to stop bleeding, and they become active during the healing process. Platelets are the smallest size and lightest among the blood cells, making them easily pushed away from the flow of blood and towards the wall of the blood vessel. Messages are sent to platelets when a blood artery in the body is injured. Thereafter, platelets rush to the damaged area and form a plug (clot) to stop the bleeding. Adhesion is the process in which a substance spreads across the surface of a damaged blood vessel to halt bleeding. This is because platelets develop sticky tentacles that help them stick (adhere) to one another when they reach the injury site. Meanwhile, chemical signals are also sent out to recruit additional platelets. Aggregation is the process through which more platelets build up on the clot, resulting in the release of growth factors and the onset of the healing process. PRP therapy is 100% safe and works wonders on fine lines, wrinkles, scars, UV damage, and dull, worn-out skin, face, neck, back of hands, scalp, and other body parts. PRP secretes a variety of growth factors that play a role in skin regeneration. PRP may also stimulate the activation of fibroblasts, causing collagen and other matrix components to be synthesized, thereby renewing the skin. PRP can be used on any portion of the body that requires special attention, and it contains growth factors that aid in natural skin healing and collagen creation. These events make the skin appear younger and more radiant. This is a natural method of anti-aging as it employs bodily materials and produces gradual and non-invasive outcomes (Ganceviciene et al., 2012). Although PRP is frequently used in clinical dermatology, there is little experimental research that validates its effects on aged fibroblasts (Kim et al., 2011).

Since RMT in aesthetic applications is more attractive compared to its other potential in disease treatments, further innovations and developments specifically aiming to restore youthful features or anti-aging conditions have been attempted.

## 2 Benefits of Regenerative Medicine Therapy

One of the benefits of RMT is increased functionality. When the tendons and tissues on and around the joints are strengthened, the range of motion in the joints is increased, allowing them to move freely and perform daily duties again (Benjamin et al., 2008). Next is faster recovery, where growth factors utilized in regenerative medicine aid in the regenerative process of tissues and tendons (Dzobo et al., 2018). Furthermore, RMT is beneficial in the treatment of cardiovascular diseases. Cardiovascular diseases can deplete oxygen supply to the heart tissue, causing scar tissue to develop while altering blood pressure and blood flow (Mozos 2015). Due to the production of various growth factors, stem cells from adult bone marrow can develop into cell types that are required to repair blood arteries and the heart (Zakrzewski et al., 2019). There are a few benefits of stem cells (HSCs and MSCs), where HSCs can make up all types of blood cells while MSCs can generate bone, bone marrow, and adipocytes (Charbord 2010). Adult stem cells that can be found in the brain can differentiate into neuron cells and non-neuron cells (Maldonado-Soto et al., 2014). Several studies have shown that hematopoietic stem cells are widely used in clinical trials (Bryder et al., 2006; Zakrzewski et al., 2019; Kabat et al., 2020; Khaddour et al., 2021).

## 3 Known Regenerative Medicine Therapy Technology Around the World

Cell therapy is one of the regenerative medicine therapy technologies that have been used worldwide. There are two types of cell therapy, which are stem cell therapy and somatic cell therapy. These cell therapies can either be autologous or allogeneic. In allogeneic therapies, a donor is a different person, who is usually a close family member or relative. It could also potentially come from a genetically matched donor. On the other hand, autologous treatments involve cells isolated from the donor. For acute myeloid leukemia (AML) patients, treatment is usually with allogeneic hematopoietic stem cell transplantation (HSCT). The treatment aims to identify prognostic factors associated with poor outcomes. The survival outcomes for Malaysian AML patients treated with allogeneic HSCT were good, where the overall 10-years overall survival (OS) and disease-free survival (DFS) for the patients after allogeneic HSCT were 63 and 67%, respectively (Ganceviciene et al., 2012). This treatment should be considered the standard therapeutic approach. In addition, stem cells have also been used in dentistry to treat periodontal disease. Gene therapy is the tissue engineering method that has been performed in periodontology (Chatterjee et al., 2013). Another RMT that has been used globally is graphene. Graphene has been employed as a scaffold to mediate stem cell growth and differentiation, whereas other researchers utilized the RMT to successfully transplant MSCs and guide their development into specific cells (Bryder et al., 2006; Kabat et al., 2020; Khaddour et al., 2021).

Numerous cell therapy trials are ongoing in Europe, the United States, and other parts of the world (Blasimme and Rial-Sebbag 2013). The regulatory framework for development and compliance procedures for the production and commercialization has progressed alongside the establishment of industries. These measures were taken to provide guidance and legislation on the assessment of safety, quality, purity, potency, and efficacy of cell therapy trials based on the experience gained from conventional pharmaceuticals and blood banks. Nevertheless, since the cell is an active therapeutic agent, the regulatory framework for such products is significantly more complex compared to typical molecular medicines, where the final product differs from cell culture bioprocessing.

The European Medicines Agency (EMA) has recommended Holoclar for approval in the European Union as the first advanced therapy medicinal product (ATMP) using stem cells (EU) (Yu et al., 2018). In adults, Holoclar is a treatment for limbal stem cell deficit (LSCD) caused by physical or chemical burns to the eye (s). It is the initial treatment option for LSCD, a rare eye disease that can lead to blindness. Stem cells have the potential to serve as the repair system of the body. Limbal stem cells are found on the edge of the cornea (the clear front section of the eye) and the sclera (the white region of the eye). LSCD can be caused by the loss of these stem cells as a result of physical or chemical damage. The repair system of the body could be aided by stem cells. Specifically, the limbal stem cells are necessary for the corneal epithelium to regenerate and repair after damage.

## 4 Regenerative Medicine Therapy in Asian Countries

Compared to Western countries, Asia is less advanced in terms of RMT developments and applications. However, it is worth noting that some Asian countries, such as Japan and Singapore are among the high-ranked countries in terms of RMT.

Table 1 shows the RMT in Asian countries with a detailed explanation of the treatment cost, success rate, and first documented dates.

**TABLE 1:** Regenerative medicine therapy in Asian countries.

Country	Type of RMT	Treatment	Cost	Success Rate	First Documented Date	References (s)
Japan	Stemchymal	Spinocerebellar ataxia	USD 5,862–58,620	Not stated	Not mentioned	Awatin and Milne, (2021)
	Allogeneic adipose-derived stem cells (eASC) injected intralesionally	Complex perianal fistulas in patients with Crohn’s disease	USD 4,000–8,000	Not stated	Not mentioned	(Lu et al., 2019; Awatin and Milne, 2021)
China	Gene therapy (Gendicine and Oncorine)	Neck cancer	USD 387 per injection	30–40% complete response and 50–60% partial response with a total response rate of 90–96%	Gendicine: 2003	Awatin and Milne, (2021)
					Oncorine: 2005	
South Korea	Hearticellgram-AMI (therapeutic stem cells)	Myocardial infarction	USD 169,202	Not stated	2011	Awatin and Milne, (2021)
	Cupistem (MSC)	Reduce	USD 3,000–5,000 per treatment	Not stated	2012	(Ramezankhani et al., 2020; Awatin and Milne, 2021)
		Inflammation and regenerate				
		damaged joint				
		tissues (Crohn’s Fistula)				

USD, united states dollars.

### 4.1 Japan

In Japan, a few RMTs have been used in treating diseases, such as Stemchymal and allogeneic adipose-derived stem cells (eASC) injected intralesionally. The most popular one is eASC since it has been used to treat complex perianal fistulas in patients with Crohn’s disease. Stemchymal is a rare treatment used for spinocerebellar ataxia given the lack of a specific therapeutic agent to cure this disease. Adipose-derived stem cells (ASCs) are a type of multipotent and malleable adherent cell generated by digesting white adipose tissue with collagenase. ASCs, which have MSC properties, have been investigated intensively in recent years (Tsuji et al., 2014). ASCs have been shown to have a high stemness, allowing them to differentiate into lineages such as osteogenic, chondrogenic, neurogenic, or myogenic. The majority of current research focused on either their ability to replace bone marrow as a readily available and plentiful source of MSCs or their use in regenerative and reconstructive medicine (Han et al., 2019). MSCs generated from adipose tissue have been particularly successful in investigations, revealing their abundance and accessibility as sources of these cells. Furthermore, there is an increasing number of clinical techniques utilizing ASCs. This highlights the need for research into the molecular bases of their activity, both *in vivo* and *in vitro*, as well as their potential interactions with cells or tissues in patients’ bodies (Jankowski et al., 2020).

### 4.2 China

In China, a wild type-p53 expressed by a recombinant adenovirus (rAd-p53) is used to treat individuals who have malignancies with mutant p53 genes (Yuan et al., 2016). In 2003, China was the first country to approve Gendicine, the first gene therapy product for clinical use in humans (Zhang et al., 2018). In various clinical applications or studies, Gendicine treatment has produced 30–40% complete response (CR) and 50–60% partial response (PR) (Xia et al., 2018). This amounted to total response rates (CR + PR) ranging from 90 to 96%, consistent with the results of phase II and phase III clinical trials on Gendicine that formed the basis for CFDA approval (Xia et al., 2018). To date, fever, arthralgia, and myalgia have been recorded as the most prevalent side effects of Gendicine treatment. Fever ranging from 37.5 to 39.5°C remained for a few hours in 50–60% of treated patients within 24 h of receiving Gendicine. Meanwhile, the cost for Gendicine is $387 per injection.

Recombinant human adenovirus type 5 (rAd5-H101) is another important RMT in China. The RMT is the first oncolytic virus licensed by the CFDA, which was commercially launched under the brand name, Oncorine, in November 2005 by Shanghai Sunway Biotech. Following phase III of the clinical trial, it was first approved for the treatment of patients with last-stage resistant nasopharyngeal cancer in combination with chemotherapy. The loss of Tp53 gene function has been associated with chemotherapy resistance and a lower survival rate in patients with non-small cell malignancies of the breast, colon, lung, head and neck, and ovaries (Nemunaitis et al., 2000). Hence, TP53 is thought to be a promising target for gene therapy in NSCC-related malignancies. The E1B-55 KD gene, which is responsible for p53 inactivation, has been completely depleted in the Oncorine adenovirus. The Oncorine only replicates in P53-deficient cancer cells, whereas adenoviruses lacking the E1b-55 KD do not replicate in normal cells. Adenoviruses release and infect nearby cells once cancer cells are lysed, triggering a cascade of the Oncorine-mediated cell cytotoxicity (Bressy et al., 2017). However, no available data states the cost for Oncorine injection.

### 4.3 South Korea

Cellgram-AMI and Cupistem are the two stem cell therapies reported in South Korea. The Korean Ministry of Foods and Medication Safety approved “Cellgram-AMI” as the world’s first stem cell drug in July 2011. HearticellgramR-AMI' was the name of the item at the time of acceptance. It is made from mesenchymal stem cells obtained from autologous bone marrow. It is a safe and effective way to increase ejection fraction in patients who have experienced acute myocardial infarction and are subjected to coronary angioplasty within 72 h of the onset of chest discomfort. The cost of Hearticellgram-AMI is about USD 3,000–5,000 per treatment (Yang 2011).

Cupistem is an autologous adipose-derived mesenchymal stem cell therapy for Crohn’s fistula that reduces inflammation and regenerates damaged joint tissues. It was approved by the Korean Ministry of Food and Drug Safety (MFDS) as an adipose tissue-derived mesenchymal stem cell (ASC) product for the first time in the world in January 2012, and its medical insurance pricing was given by the Health Insurance Review & Assessment (HIRA) Service in January 2014. The most notable aspect of this medicine is its capacity to maintain therapeutic efficacy over time. Accordingly, 82% of patients with complex Crohn’s fistulas that used Cupistem were reported to be completely healed by week 8 after the treatment, and 81% had a sustained response by week 96 (Sands et al., 2004). The remarkable long-term efficacy of the product was attributed to the immunological modulation impact of Anterogen-derived ASCs.

## 5 Regenerative Medicine Therapy in Malaysia

In Malaysia, regenerative medicine has gained more interest over the years. More researchers have been exploring the possibility of utilizing RMT in Malaysian clinical settings as an alternative to conventional treatment or to possibly become the superior choice of treatment in the future. In comparison to other developed nations, Malaysia is still far behind in keeping up with the current advances in medical therapies. Nonetheless, numerous developments have been recorded in Malaysia’s research sector in investigating RMTs and their clinical implications.

Table 2 presents detailed information on RMTs in Malaysia, which includes the related diseases, treatment cost, and the first documented dates.

**TABLE 2:** Regenerative medicine therapy in Malaysia.

Type of RMT	Related disease	Cost	First Documented Date	References (s)
Stem cell (Hematopoietic stem cells)	Blood cancer (leukemia, lymphoma)	Private hospitals: MYR 200,000–250,000	1987	(Gan et al., 2008; Ullah et al., 2015)
		Government hospitals: MYR 40,000–60,000		
Stem cell	Anti-aging and diabetes	MYR 40,000–60,000	No date mentioned	Ministry of Health, (2012)
Stem cell (Mesenchymal stem cell)	Autoimmune diseases such as Alzheimer’s disease (AD) and Parkinson’s disease (PD)	MYR 40,000–60,000	No date mentioned	Ministry of Health, (2012)
Platelet-rich plasma	Wound healing in trauma and joint injuries	MYR 800–2,000	2013	Jaafar et al. (2017)

In private medical centers, the average cost for using hematopoietic stem cells (HSC - Hematopoietic Stem Cells) as an RMT for blood cancer (leukemia and lymphoma) is in the range of MYR 200,000 to 250,000. Through the Medical Assistance Fund of the Malaysian Ministry of Health Malaysia (MOH), the cost of treatment can be reduced to around MYR 40,000 to 60,000 at all University Hospitals. In addition to its use for the treatment of blood cancer, stem cells are still considered experimental and are only allowed for medical research purposes. However, many private centers provide stem cell treatments for other uses, such as for the treatment of knee joints, diabetes, and anti-aging. Since the use of RMT is still experimental as regulated by the MOH, the experimental type of stem cell treatment is not paid for or covered by the insurance company. Apart from bone marrow transplantation, stem cell treatment for other causes is already being investigated in clinical trials and has shown promising outcomes for treating disorders, such as diabetes, heart disease, knee osteoarthritis, autism, and anti-aging (Loo and Wong 2021). Approximately, 100 million MSCs treatment in Malaysia might cost between MYR 50,000 and MYR 80,000. Malaysia is among the countries in Southeast Asia in which PRP is extensively used for face, neck, and scalp treatments.

A few clinical trials are currently being conducted in Malaysia and have not been used yet in medical applications. Table 3 shows a few clinical trials of RMT that are currently ongoing in Malaysia.

**TABLE 3:** Current regenerative medicine therapy (RMT) in clinical trials.

Current RMT Clinical Trials	Treatment	Update on the Clinical Trial	References (s)
Precision medicine	Treating cancer through a tumor profiling approach that can identify various anti-cancer-therapies	No data mentioned	(Malone et al., 2020; Lightner and Chan, 2021)
Unmatched donor umbilical cord-derived mesenchymal stem cells	Knee articular cartilage defects	Not yet recruiting	(Wu et al., 2019)
Bone marrow, hematopoietic stem cell (HSC) transplants	Blood diseases and cancer	No data mentioned	Khaddour et al. (2021)
Cellular therapy	Ankle sprain	No data mentioned	Loo and Wong, (2021)
Autologous peripheral blood stem cell	Articular cartilage regeneration	Completed	Saw et al. (2013)
Mesenchymal Stem Cell	Anti-aging	Phase 1 (Not recruiting)	Zarei and Abbaszadeh, (2019)
Sirolimus-eluting Iron Bioresorbable Coronary Scaffold System	Single Coronary Vessel disease	Recruiting	Zheng et al. (2019)

Several RMTs are currently in clinical trials in Malaysia with precision medicine being one of them. Precision medicine can be used to treat cancer through a tumor profiling approach that can identify various anti-cancer-therapies (Krzyszczyk et al., 2018). Precision medicine is a “developing approach to illness treatment and prevention that considers an individual’s genetic diversity, environment, and lifestyle.” Doctors and researchers will be able to accurately predict whether treatment and prevention methods for a specific disease will be successful in the groups of people subjected to this approach. It differs from a one-size-fits-all strategy in which disease treatment and prevention techniques are designed for a specific person with little regard for individual differences. Precision medicine in cancer uses unique information about an individual’s tumor to help in the diagnosis, treatment planning, monitoring treatment effectiveness, and prognosis (Malone et al., 2020; Lightner and Chan 2021). Examples of precision medicine are targeted therapies for specific types of cancer cells, such as HER2-positive breast cancer cells. This approach plays the role of tumor marker testing to aid in cancer diagnosis.

Generally, cancer can be treated using chemotherapy, radiation, and surgery. These methods involve the application of medical tools by doctors while treating cancer. Meanwhile, in precision medicine, the doctors employ a patient’s genes to uncover clues for cancer treatment.

Unmatched donor umbilical cord-derived MSCs are also believed to treat knee articular cartilage defects (Arshi et al., 2020), and are currently viewed as an extensive research area for stem cells. However, their clinical applications are not common in Malaysia given the limitations of evidence-based clinical trials, particularly in hospitals with limited resources and competence. These RMTs are not yet recruiting and could not be used for medical purposes. Another example of a clinical trial conducted in the joint research in gene therapy for *β*-thalassemia. *β*-thalassemia is a blood disorder in which hemoglobin production is deficient in adults. Clinical trials have shown that by splitting certain genes, patients undergoing treatment can produce sufficient hemoglobin to reduce the effects of their disorders.

Another study focused on the treatment for Spinal Muscular Atrophy (SMA), a genetic disease that affects the part of the nervous system that controls voluntary muscle movement (Khaddour et al., 2021). The study found that patients suffering from SMA either lost or had a mutated gene known as the Survival Motor Neuron 1 (SMN1) gene. Missing or mutated genes can be replaced using viral transport mechanisms to insert corrected genes. Based on the observational studies conducted using mice models, the infusion of healthy bone marrow components into a myelosuppressive bone marrow could stimulate recovery of function in the recipient (Khaddour et al., 2021). A pediatric patient with severe combined immunodeficiency syndrome received the first successful allogeneic bone marrow transplant in Minnesota in 1968 (Khaddour et al., 2021). Allogeneic and autologous stem cell transplantation has become more popular in the United States and worldwide since then. The process of transplanting cells to an injured location to heal or replace damaged tissues or cells is known as cellular therapy. Several studies have demonstrated the introduction of stem cells using cellular treatment techniques. However, these studies only provide preclinical proof while clinical evidence in this area is sparse. Although little research has been performed on the cells of the ankle ligaments, researchers believed that they have distinct cellular features and components than the other ligaments, which makes it challenging for cellular therapy to restore the wounded ligaments to their pre-injury form. Another research that is still in the phase of a clinical trial is autologous peripheral blood stem cells (Saw et al., 2013). This autologous peripheral blood stem cell is used for articular cartilage regeneration and the update of these stem cells has been completed (Saw et al., 2013). Presently, the clinical trial is at the second phase and it has to pass through the third phase before completion. Approval from the governments of the countries is required before the stem cell can be used.

Another clinical trial uses MSCs as an anti-aging. The sources of MSCs are either autologous (adipose tissue) or allogeneic (adipose tissue or umbilical cord) (Zarei and Abbaszadeh 2019). This research is to evaluate the safety and efficacy of human MSCs infusion therapy in preserving general wellness (Saw et al., 2013) and reversing the effects of aging in the study population. The research is still in phase 1 clinical trial and is not yet recruiting. Furthermore, the Sirolimus-eluting Iron Bioresorbable Coronary Scaffold System (IBS) is used to treat single coronary vessel disease (Zheng et al., 2019). This study was mainly designed to evaluate the feasibility, preliminary safety, and efficacy (IBS) and this research are currently still recruiting (Zheng et al., 2019).

## 6 Most Prominent Regenerative Medicine Therapy in Malaysia

Some RMTs such as stem cells and PRP have been used in clinical settings in Malaysia. These RMTs have been used widely and most are offered in government and private hospitals in Malaysia. The cost of the treatments is quite expensive, especially in private hospitals. Table 4 highlights the most prominent RMTs in Malaysia, the hospitals offering RMT, and the costs involved.

**TABLE 4:** Most prominent regenerative medicine therapy in Malaysia.

RMT	Hospital/type of Treatment	Cost	References (s)
Stem cell therapy (MSC and HSCs)	Hospital Ampang Clinical Stem Cell Services (Blood cancer)	MYR 50,000 to MYR 70,000 for 100 million cells	Ministry of Health, (2012)
Platelet-Rich Plasma (PRP)	HUSM/knee joint pain	• PRP treatment for face/wrinkles: MYR 700–1,500/session	Chang, (2021)
		• PRP injection for knees/joint pain: MYR 3,000–4,000/session	
		• PRP hair treatment: MYR 2,500–3,500/session	
Cell therapy	• UKM Medical Centre (UKMMC)	MYR 50,000 to MY R80,000	Ministry of Health, (2012)
	• Hospital Ampang Clinical Stem Cell Services, autoimmune disease, urinary problems, and infectious disease		

MSCs and HSCs are the most commonly used stem cells in RMT in Malaysia. One of the best hospitals that offer stem cell therapy is Hospital Ampang Clinical Stem Cell Services, which focuses more on blood cancer treatment. According to the MOH, the treatment cost is about MYR 50,000 to MYR 70,000 for 100 million cells. The cost in government hospitals is quite cheap compared to private hospitals. These stem cells are mostly used in treating blood cancer, such as leukemia. MSCs have been widely used in regenerative medicine for bone regeneration and are mostly still investigated in clinical studies. MSCs appear to be promising targets for cell treatment in a range of malignancies (Lee et al., 2019).

In Malaysia, PRP treatment is rare but the treatment is practiced, especially in Hospital Universiti Sains Malaysia (HUSM). The cost of PRP treatment in Malaysia ranges from MYR 800 to MYR 1500 per treatment. Nevertheless, the cost varies depending on the location and popularity of the hospital, and the PRP preparation kit used. The PRP treatments in Malaysia are mostly utilized to treat osteoarthritis. Osteoarthritis is the most common cause of joint discomfort and a condition that primarily affects the elderly. Osteoarthritis can affect every joint, but the knees, hips, shoulders, thumbs, and spine are the most commonly affected. The shock absorbers in these joints are called cartilage, and they are located within the joint. These cartilages are vulnerable to wear and strain as one gets older, and the process is accelerated by injury or misuse of the joints. PRP is useful in the treatment of osteoarthritis-related joint discomfort. It is thought that injecting PRP into the afflicted joints will induce cartilage formation. The pain caused by bone friction will be substantially decreased with the regeneration of these natural cushions. PRP has several advantages over surgical replacement, including being a non-invasive therapy with far lower risks. Surgical interventions expose a patient to infection, blood loss, anesthetic chemical allergies, and surgery failure that necessitates revision. The plasma-rich platelet obtained from the patient’s blood is another benefit of PRP, indicating that the material will not be rejected by the body as foreign and no reaction will occur. The platelets are extracted from the patient’s blood via centrifugation.

The application of PRP for cell therapy is seen in autoimmune diseases, urinary problems, and infectious diseases. In Malaysia, cell therapy may cost MYR 50,000 to MYR 80,000. The Cell Therapy Center is a Center of Excellence at UKM Medical Centre (UKMMC) that provides cell-based therapy to patients with blood disorders, cancers, bone marrow diseases, and degenerative diseases, including HSCs, lymphocytes, dendritic cells, MSCs, and mononuclear cells. The Cell Therapy Centre founded in November 2006, aspires to be a world leader in cancer immunotherapy and regenerative medicine research and development (R&D). In July 2008, a brand-new transplant unit with 10 transplant rooms, 6 post-transplant rooms, and 2 suites was opened. The Stem Cell Transplant unit was previously located in Hospital Kuala Lumpur, but it was relocated to Hospital Ampang in 2006, and designated as the National Referral Centre for Hematological Expertise. Since 2010, Hospital Ampang has been able to perform 180 transplants each year compared to only 40 to 45 transplants conducted earlier. The waiting period is now less than 2 months (compared to 6 months when the program first began), owing to the time required for donor and patient evaluations. Once a donor is found, emergency cases such as bone marrow failure can be treated within 2 weeks. From 1999 through 25 October 2012, this unit performed 834 adult transplants, including 443 autologous transplants, 359 allogeneic-fully matched sibling transplants, 18 allogeneic-matched unrelated transplants, and 7 cord blood transplants (Ministry of Health 2012).

The Malaysian Stem Cell Therapy Working Group (SCM) is a network of multi-disciplined medical professionals and specialists who provide sophisticated, research-driven regenerative medicine, integrative medicine, and lifestyle medicine in the country. In the case of stem cell therapy, there is no one-size-fits-all option. Mid Valley City, Pantai Hospital Kuala Lumpur, Subang Jaya Medical Centre, Life Care Medical Centre, Assunta Hospital, Publika KL, Petaling Jaya, Shah Alam, and Kajang are among the Malaysian Stem Cell nine partner clinics in the Klang Valley (Ministry of Health 2012). However, no available data to depict the number of treatments performed in these partner clinics. HUSM has specialists who have studied PRP abroad and have been practicing this treatment since 2018. They also ensure that all the equipment needed to provide PRP is ready to be used in surgery. This hospital has developed a new technique for extracting platelet-rich plasma from autologous white blood cells with a PRP enrichment of 6–10 times, which is about 3–5 times higher than for conventional ones. The treatment has been proven to be more effective at rejuvenating the skin, removing wrinkles, and reducing abnormalities caused by acne marks. Likewise, there is no data to reflect the number of treatments (PRP) administered in the center.

## 7 Malaysian Regulation on the Use of Regenerative Medicine Therapy

Regulatory oversight on the use of these biotechnology-derived cells and tissues as therapeutic products for the treatment of diseases is important to prevent any potential serious attendant risks to public health. Drug regulatory agencies across the globe including the United States, Canada, the European Union, Singapore, Japan, and Korea recognize cell therapy products as drugs or medicinal products. Regulatory agencies also recognize the current drug regulatory framework, which might not be the best framework to regulate these products. Hence, drug regulatory agencies have either established or are developing a separate framework to better regulate cell therapy products in the last few years (Kellathur and Lou 2012). As shown in Table 5, different countries worldwide have various regulations and laws that specify the clinical applications and research of RMT as of 2010.

**TABLE 5:** The regulation of RMT in clinical application and research according to countries.

Status of Regulation	Countries
Regulated by Law	Australia, Belgium, China, Denmark, Finland, Hungary, India, Israel, Japan, Singapore, Spain, South Korea, Sweden, and the United Kingdom
Law in preparation	Brazil, Canada, France, Iran, Netherlands, South Africa, Portugal, Spain, and Taiwan
Prohibited	Austria, France, Germany, Ireland, Italy, Netherlands, and Norway
No law yet	Africa, Belgium, Czech Republic, Greece, Italy, Poland, Slovenia, Switzerland, Turkey, and Malaysia

In Malaysia, there is no law with regards to RMT, either in clinical application or research. Meanwhile, only a general regulation and guidelines for the use of stem cells in research and therapy exist, which was documented in 2009 (CRC 2009). Only hematopoietic stem cell and umbilical cord stem cell transplantations are allowed in Malaysia, and they have become the most established form of stem cell therapy in the country. The use of other stem cells such as hESCs and somatic stem cells is considered experimental. Xenotransplantation or therapies involving the use of animal stem cells or animal cells are currently prohibited. Private healthcare facilities and services intending to perform or perform stem cell or cell-based therapies need to be licensed under the Private Healthcare Facility and Services Act 1998 (CRC 2009). The guidelines provided were mainly based on those used by the United States Food and Drugs Administration (US FDA). Hence, it is time for Malaysia to have specific legislation for the application of RMT, either for aesthetic use or for disease treatments. Such legislation will help to regulate and monitor proper applications of RMT in the country, as well as prohibit any misuse or mis-advertising of RMT that is harmful to the public.

**FIGURE 2:**
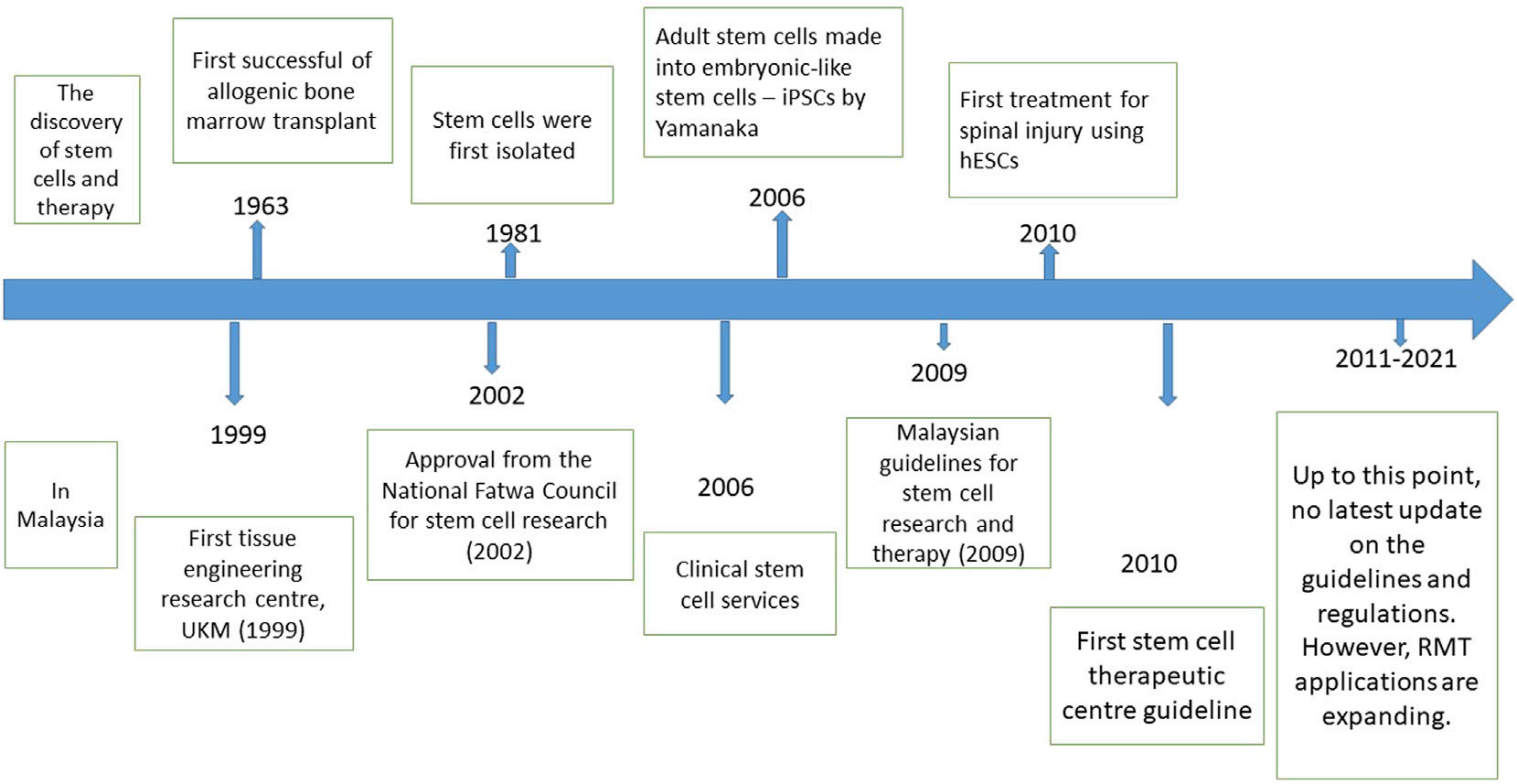
(Top) The timeline of the most significant discovery of stem cells and therapy worldwide. (Bottom) The timeline of RMT development, including the regulations and fatwa involving stem cell research in Malaysia.

Another guideline in Malaysia regarding cell and gene therapy products (CGTPs) was documented in 2021 by the National Pharmaceutical Regulatory Agency (NPRA). However, this guideline is too brief as it only specifies that facilities producing CGTPs will only need to comply with the good manufacturing practice (GMP) that has been established. A more detailed and focused guideline is necessary to ensure a higher standard for producing CGTPs as it involves handling live cells, which will be used for various severe and chronic illnesses. Figure 2 illustrates the development of RMT, including but not limited to stem cells therapy worldwide and in Malaysia.

## 8 Cultural and Religious Barriers Against Regenerative Medicine Therapy Application in Malaysia

Lack of information and awareness regarding RMT are expected among the public as it is a newly developed technology. Hence, there are different views on cultural and religious aspects towards RMT. There may also be some cultural and religious barriers against RMT application, specifically in Malaysia.

Malaysia is a multicultural and multi-religious country that is proud of the diversity among its people. Malaysia’s population is currently 32.37 million people with 61.3% of the population practicing Islam, which amounts to approximately 19.5 million people (Ahmad 2007), followed by 19.8% Buddhism, 6.3% Hinduism and 9.2% Christianity (Ahmad 2007). Such diversity in beliefs may pose some challenges in implementing RMT for clinical applications, especially if the religion opposes it.

With the majority of believers in Malaysia, Islam states that the sources of stem cells must be from adult stem cells, the procedure does not cause harm, and the fetus is aborted spontaneously or miscarried. The National Fatwa Deliberation Committee for the 51st time on 11 March 2002 (Mazri and E-fatwa 2012) has decided as follows in determining the use of stem cells: 1) human cloning for any purpose whatsoever is illegal because it is contrary to the nature of the event of man who has been ordained by God, 2) the use of stem cells for medical purposes in a study which does not involve the cloning process is required as long as it does not conflict with the Sharia law. On 25 May 2006, the Mufti Department of Selangor also stated that stem cells from specified sources are to be used for treatment purposes, medicine, and survey through fatwa legislation no. 13. The sources of stem cells are specified as follows: 1) from an adult (adult stem cell) with permission and the procedure did not cause harm, 2) from a child with the parent’s consent and the procedure did no harm, 3) from the placenta and umbilical cord blood of the baby with consent from parents, 4) from a spontaneous abortion or miscarriage as a result of medical treatment permitted by Sharia provided that the parent’s consent is obtained, not a fetus that is intentionally aborted or aborted without medical reasons permitted by Sharia, 5) from excess embryos stored frozen from IVF fertility assistance technology with the condition of obtaining the parent’s consent.

On the other hand, according to Christianity, the cloning process demands the use of human embryos in which cells can be produced to make new organs to obtain the necessary DNA where some embryos must be killed (Ghazali et al., 2014). Meanwhile, Buddhism stated that embryogenesis at 4–5 days post-fertilization is not categorized as a living thing. According to Hinduism, there are three types of living things: plants, animals, and humans (Sivaraman and Noor 2017). The human body, including all its elements, also has a nature of its own. The same applies to nails on the feet, hair on the head, and so on. All of that goes according to the law of nature or the law of omnipotence (Sivaraman and Noor 2017). Table 6 summarizes the religious views towards RMT in Malaysia.

**TABLE 6:** Cultural barriers against regenerative medicine therapy and local views on regenerative medicine therapy in Malaysia.

Religion	View towards RMT	References (s)
Islam	Sources of stem cell: adult stem cell, the procedure does not cause harm. The fetus is aborted spontaneously or miscarried	Mazri and E-fatwa, (2012)
Christian	The cloning process demands the use of human embryos; cells can be produced to make new organs to obtain the necessary DNA; some embryos must be killed	Ghazali et al. (2014)
Buddhist	Embryogenesis at 4–5 days post-fertilization, not in the category of living things	Sivaraman and Noor, (2017)
Hinduism	Three types of living things: plants, animals, and humans. The human body, including all its elements, also has a nature of its own	Sivaraman and Noor, (2017)

In addition to religious beliefs, Malaysia also has various cultures that go back to their ancestral practices, especially among the indigenous people, such as Iban, Kadazan, Kayan, and many more. Each ethnic group will have its own cultures that are still practiced today. The views towards RMT according to local cultures have received extensive attention in terms of ethical issues in regenerative medicine. These topics have consumed significant debate about the use of human embryonic stem cells in research, specifically, the destruction of embryos in order to create cell lines. Others include the disputes over the role of federal funding for research with human embryonic stem cells, and concerns about the safety, scientific purity, and adequacy of consent for the use of human embryonic stem cell lines in research. Like other novel biotechnologies, regenerative medicine raises several additional critical research ethics issues that are not new but have not yet been adequately addressed. Instead, as is common in new science, familiar and longstanding questions appear in the fresh context of regenerative medicine research and renew old debates (McCormick and Huso 2010). More studies are needed to fully understand the perspective of the local cultures and religious beliefs towards RMT applications in Malaysia.

## 9 Limitations

Further basic studies are required to completely comprehend the process of deep cell specialization in humans. Scientists are currently striving to develop a reproducible procedure for transforming stem cells into the cells and tissues needed for transplantation. Furthermore, scientists must overcome the problem of immunological rejection before mature cells produced from embryonic stem cells or hematopoietic stem cells may be employed in transplantation. Incompatibility between these cells must be kept to a minimum as they are genetically distinct from that of the receiver. Adult stem cell research has also faced numerous challenges, including identifying, isolating, and detecting specific cells, developing adult stem cells in the lab, and demonstrating adaptability (McCormick and Huso 2010). Several studies are currently ongoing in this sector all over the world, and several trials are being performed in the clinic (Hall et al., 2010). For instance, one of such research is the successful implantation of artificial bladders into young children (Atala et al., 2006), and a trachea made from a patient’s split trachea and seeded with autologous MSCs, which has been successfully transplanted back into the same patient (Macchiarini et al., 2008). Most RMTs in Malaysia are yet to be approved by the government as they are currently undergoing clinical trials.

Stem cells and cosmetic medicine are other areas of technology that are also affected by medical tourism. Given their unique regenerative quality, stem cells are regarded as an emerging technology for their medicinal potential. The ability of stem cells to self-renew, duplicate, and differentiate into any cell or tissue has paved the way for a slew of exciting new medicines and research that would otherwise be unavailable in many parts of the world (Bianco et al., 2013; Gopalan et al., 2020). It persuades people to fly abroad in search of these procedures, expecting to be cured or treated, despite the risk of being exploited by unproven therapies (Zakrzewski et al., 2019). The practice of traveling to other countries with lax or no regulations in search of experimental or untested stem cell therapy is known as “stem cell tourism,” and it is a subset of medical tourism in this study. This is also known as health tourism, which will invalidate and reduce the availability of sufficient data for RMT in Malaysia for detailed comparisons with other hospitals. Some treatments (RMT) are conducted in private hospitals where the staff or organization does not report the data of RMT performed, thereby limiting the data collection and comparison with the government hospitals. Some Malaysians will find a hospital that has advanced technology in other countries to treat the disease. This might be one of the limitations of RMT in Malaysia following the limitation in the data obtained.

Many research projects on stem cells have lately been allowed in Malaysia. However, they are still in the early stages of development due to a lack of funds, specialized equipment, and skilled personnel. These limitations might explain why RMT is not yet widely known in Malaysia. Research on stem cells could be easily interrupted when funds are lacking. Skilled personnel is needed to perform stem cell research and increase the use of RMT in medicine to treat diseases.

## 10 Prospect of Regenerative Medicine Therapy in Malaysia

One of the currently ongoing research projects that shows potential and great promise in clinical applications of RMT in Malaysia is therapeutic cloning. Therapeutic cloning, also known as somatic cell nuclear transfer, is a method for producing versatile stem cells without the need for fertilized eggs. The nucleus, which contains the genetic material, is removed from the unfertilized egg. Furthermore, the nucleus of a donor’s cell is also removed. The donor nucleus is injected into the egg to replace the exteriorized nucleus in a procedure known as nuclear transfer. Allowing the egg to divide results in the formation of a blastocyst. This procedure produces a line of stem cells that is genetically identical to the donor’s cells. Some researchers believe that stem cells obtained by therapeutic cloning have advantages over those derived from fertilized eggs. This is because cloned cells are less likely to be rejected once transplanted back into the donor and may allow researchers to precisely examine how a disease develops. Notably, this research has not yet been successful and is still ongoing. In recent investigations, researchers have modified the therapeutic cloning technique to create human pluripotent stem cells. Researchers are still looking into the possibility of human therapeutic cloning.

Regenerative medicine therapy in Malaysia has numerous potentials to be developed and applied in clinical settings. These RMTs can be used to treat diseases, especially fatal chronic diseases. After decades of research, stem cell therapy is proving to be a tremendous game-changer in medicine. The capacity of stem cells is increasing with each trial, yet there are still numerous challenges to solve. Nevertheless, stem cells have a huge impact on regenerative medicine and transplantation. Untreatable neurodegenerative illnesses might be successfully treated using stem cell therapy in the near future. The use of a patient’s own cells is possible thanks to induced pluripotency. Tissue banks are gaining popularity as they collect cells that can be used in regenerative medicine to fight present and future ailments. Medical practitioners and scientists are better able to extend human life than at any other point in history owing to stem cell therapy and all of its restorative effects (Zakrzewski et al., 2019).

## 11 Conclusion

There are many more technological advances to be explored in RMT and applied in Malaysian clinical settings. Numerous ongoing research and clinical trials have shown the potential of clinical applications of RMT in Malaysia. The most popular and well-known RMT in Malaysia is cell therapy. Mesenchymal stem cells (MSCs) are widely used in the country to treat chronic diseases while MSC-based iCell treatments are being studied in several clinical trials. MSCs are heterogeneous and numerous sources are employed to separate and create these cell populations for clinical trials. Important perspectives on the different nature of the MSCs populations in use clinically and their various sources have been critically addressed recently (Bianco et al., 2013).

Given that RMT is still relatively a new field, the results from early clinical studies need to be understood so that others can learn from them. Overall, it appears that substantial investment and funding have been injected in preclinical research and clinical trials, but only a sliver of success has been obtained thus far. On the other hand, clinical reports will continue to evolve and general trends will emerge. It is apparent that limbal stem cells have developed, neural stem cells are promising for regenerative repair, pluripotent stem cells have shown exceptional potential in regenerative medicine, and MSCs are presently the most popular cell type in clinical trials. These studies continue to receive extensive interest due to news coverage and patient expectations of significant benefits (Trounson and McDonald 2015). Several studies are ongoing in Malaysia, and the findings will depict the great potential and increase the country’s status as one of the popular countries contributing ideas on regenerative medicine to be used in clinical settings.
